# Ixazomib-induced Sweet’s syndrome: A case report

**DOI:** 10.1177/2050313X231181034

**Published:** 2023-06-16

**Authors:** Jeanne Bellemare, Hannah Laure El Fassy, Alexandra Mereniuk

**Affiliations:** 1 Division of Dermatology, Hôpital du Sacré-Coeur de Montréal, Université de Montréal, Montreal, QC, Canada; 2 Division of Immunology-Allergy, Hôpital du Sacré-Coeur de Montréal, Université de Montréal, Montreal, QC, Canada

**Keywords:** Sweet’s syndrome, ixazomib, drug-induced, multiple myeloma, case report

## Abstract

Ixazomib, a proteasome inhibitor commonly used for the treatment of multiple myeloma, is a rare cause of Sweet’s syndrome. We present a 62-year-old man who developed drug-induced Sweet’s syndrome during his fifth cycle of ixazomib for treatment of refractory multiple myeloma. Monthly rechallenge led to the recurrence of symptoms. The patient was successfully treated with addition of weekly corticosteroids and resumed his cancer treatment.

## Introduction

Sweet’s syndrome (SS) is a rare neutrophilic dermatosis characterized by an acute onset of fever, painful erythematous skin lesions on the upper half of the body, and an elevated neutrophil count. It has been associated with a number of disorders and triggers, including drugs. Management of drug-induced SS includes withdrawal of the culprit medication. However, when discontinuation of the drug is not an option, the addition of corticosteroids or antineutrophilic agents such as colchicine or dapsone may be used to hasten resolution or help control symptoms.^1^ To our knowledge, only three cases of SS induced by ixazomib, a proteasome inhibitor commonly used for the treatment of multiple myeloma (MM), have been reported to date.^2-4^ This case illustrates that the addition of oral pulse corticosteroids may allow continuation of therapy in patients with MM for whom alternatives are not the best options.

## Case report

**Figure 1.**
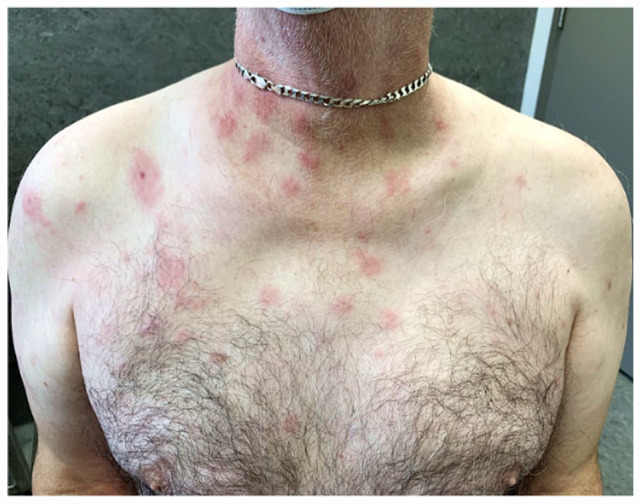
Multiple erythematous infiltrated papules and plaques on the tronc.

We present a case of a 62-year-old man diagnosed with MM initially treated by autologous hematopoietic stem cell transplant. Due to repeated episodes of pneumonia on lenalidomide maintenance, he was then switched to ixazomib (days 1, 8, and 15 monthly). Two days into his fifth cycle of ixazomib, the patient presented with a subfebrile state (37.9°C) and erythematous infiltrated papules on the trunk, neck, and face (Figure 1).

Laboratory tests were notable for neutrophilia (9.3 × 10^9^ g/L), and skin biopsies revealed mild to moderate non-specific inflammation with papillary dermal edema and occasional neutrophils.

The lesions subsided within days, but ixazomib was temporarily withheld by the oncology team, without any recurrence of lesions. Four months later, upon restarting ixazomib, the patient developed similar lesions 2 days after his first dose, with exponential progression after the second and third doses of the cycle, and gradual resolution thereafter. Monthly rechallenge led to recurrence and worsening of the number of lesions, and prednisone 0.5 mg/kg was finally added after the second half of each cycle to hasten resolution of the rash. Given the striking recurrence, lack of other convincing culprit, and rapid response to corticosteroids, a clinical diagnosis of SS induced by ixazomib was confirmed.

Given the lack of alternative treatment for the patient’s MM, colchicine was trialed as a steroid-sparing agent. Failure to control the skin after 3 months, led to retrial of a new corticosteroid regimen. Excellent control of the SS was finally achieved with pulsed dexamethasone 4 mg per day on two consecutive days after each dose of ixazomib, and was well tolerated.

## Discussion

Sweet’s syndrome (SS) is frequently subdivided into three categories: classic SS, which includes infectious and inflammatory disorders, paraneoplastic SS, and drug-induced SS. Paraneoplastic SS accounts for about 20% of all cases and is most commonly associated with hematologic malignancies, in particular acute myelogenous leukemia and myelodysplatic syndromes.^5^ Although rarely, it has also been associated with underlying MM.^6^ As for drug-induced SS, it is classically associated with G-CSF (granulocyte colony stimulating factor) and tretinoin (all-trans retinoic acid), but the list of drugs continues to grow to include several antimicrobials, antineoplastics, anticonvulsants, antipsychotics, cardiovascular agents, immunosuppressants, and more.^1^ Skin lesions usually develop within 1–2 weeks after initial exposure, but delays ranging from several months up to 2 years have been reported.^7^ Discontinuation of the causative agent leads to resolution of symptoms within days up to 1 month and relapses occur upon re-exposure. Clinical presentation and histopathology are similar to classic SS. Clinical remission can be successfully achieved with withdrawal of the culprit drug. If discontinuation of the provoking medication is not feasible, as was the case with our patient with refractory MM, addition of corticosteroids or steroid-sparing therapy such as colchicine or dapsone may be a reasonable management option.

In 1996, Walker and Cohen established the current diagnostic criteria for drug-induced SS: (1) abrupt onset of painful erythematous plaques or nodules, (2) histopathologic evidence of a dense neutrophilic infiltrate without evidence of leukocytoclastic vasculitis, (3) pyrexia > 38°C, (4) temporal relationship between drug ingestion and clinical presentation, or temporally related recurrence after oral challenge, and (5) temporally related resolution of lesions after drug withdrawal or treatment with systemic corticosteroids.^8^ The Naranjo nomogram^9^ is another useful tool for causality assessment for adverse drug events that considers several key elements lacking in the Walker and Cohen criteria, such as previous conclusive literature. In our case, it gives a score of 5 (probable), reflecting that the underlying MM cannot be excluded as a potential culprit, although much less likely. While histopathology did not show the typical dense dermal neutrophilic infiltrate of SS, the clinical presentation, the previous conclusive reports in the literature, the well-established temporal relationship between the symptoms and exposure to ixazomib as well as the rapid response to systemic corticosteroids led to a clinical diagnosis of ixazomib-induced SS.

Three other cases of confirmed ixazomib-induced SS in the setting of refractory MM have been described in the literature.^2-4^ All patients were treated with a combination of ixazomib, lenalidomide and dexamethasone, and developed their symptoms within the first or second cycle. Ixazomib was discontinued in all cases, and the patient’s symptoms rapidly resolved with topical corticosteroids (2/3) as their lenalidomide treatment was continued, which was not an alternative in our patient.

To our knowledge, we present the first case of ixazomib-induced SS that has successfully resumed treatment with the addition of corticosteroids. This case illustrates that continuation of the treatment may be an option in patients with high-risk MM.
